# Intraspecific variation in the Cambrian: new observations on the morphology of the Chengjiang euarthropod *Sinoburius lunaris*

**DOI:** 10.1186/s12862-021-01854-1

**Published:** 2021-06-21

**Authors:** Michel Schmidt, Yu Liu, Xianguang Hou, Joachim T. Haug, Carolin Haug, Huijan Mai, Roland R. Melzer

**Affiliations:** 1 Faculty of Biology, Ludwig-Maximilians-Universität München, 82152 Planegg-Martinsried, Germany; 2 MEC International Joint Laboratory for Palaeobiology and Palaeoenvironment, Yunnan University, 2 North Cuihu Road, Kunming, 650091 People’s Republic of China; 3 Bavarian State Collection of Zoology, Bavarian Natural History Collections, Münchhausenstr. 21, 81247 Munich, Germany; 4 Yunnan Key Laboratory for Palaeobiology, Yunnan University, 2 North Cuihu Road, Kunming, 650091 People’s Republic of China; 5 GeoBio-Center, Ludwig-Maximilians-Universität München, Richard-Wagner-Str. 10, 80333 Munich, Germany

**Keywords:** Intraspecific variation, Amira, 3D modelling, Volume rendering, Blender models

## Abstract

**Background:**

The Chengjiang biota from southwest China (518-million-years old, early Cambrian) has yielded nearly 300 species, of which more than 80 species represent early chelicerates, crustaceans and relatives. The application of µCT-techniques combined with 3D software (e.g., Drishti), has been shown to be a powerful tool in revealing and analyzing 3D features of the Chengjiang euarthropods. In order to address several open questions that remained from previous studies on the morphology of the xandarellid euarthropod *Sinoburius lunaris*, we reinvestigated the µCT data with Amira to obtain a different approach of visualization and to generate new volume-rendered models. Furthermore, we used Blender to design 3D models showing aspects of intraspecific variation.

**Results:**

New findings are: (1) antennulae consist of additional proximal articles that have not been detected before; (2) compared to other appendages, the second post-antennular appendage has a unique shape, and its endopod is comprised of only five articles (instead of seven); (3) the pygidium bears four pairs of appendages which are observed in all specimens. On the other hand, differences between specimens also have been detected. These include the presence/absence of diplotergites resulting in different numbers of post-antennular appendages and tergites and different distances between the tip of the hypostome and the anterior margin of the head shield.

**Conclusions:**

Those new observations reveal intraspecific variation among Chengjiang euarthropods not observed before and encourage considerations about possible sexual dimorphic pairs or ontogenetic stages. *Sinoburius lunaris* is a variable species with respect to its morphological characters, cautioning that taxon-specific variabilities need to be considered when exploring new species.

**Supplementary Information:**

The online version contains supplementary material available at 10.1186/s12862-021-01854-1.

## Background

The Cambrian marks the quite sudden diversification of animal life. Our knowledge of this very early period of the history of animals has been provided by few exceptional fossil Lagerstätten. Besides the long-known and well-studied Burgess Shale biota from Canada [1–5] or the ‘Orsten’ fossils from Sweden [6, 7], another famous but older one is the Chinese Chengjiang biota of about 518 million years in age [8]. Since its discovery in 1984, the Chengjiang biota [9, 10] has provided fossils that have been so far categorized in more than 300 formally described species, (summarized in ref. [8]), with most of them being representatives of the group Euarthropoda. Early studies have involved “traditional” methods such as light microscopy under directional reflective light, needle preparation, and *camera lucida* drawings [11]. Some improvements were achieved by more sophisticated or “structured” light [12–14] also in combination with composite imaging.

Lately, also µCT-imaging has provided promising results [15–17]. In the following years, a series of publications have shown the powerful combination of µCT-scanning and 3D rendering techniques in revealing appendage morphology of various arthropods from the Chengjiang biota [18–25]. The µCT data published in all these studies have been mostly rendered and analyzed with the public domain software, *Drishti* [26; https://github.com/nci/drishti]. Here, we choose the datasets of *Sinoburius lunaris* previously published in Chen et al. [20] and use *Amira* (https://www.thermofisher.com) and *Blender* (https://www.blender.org, following ref. [27]) to shed new light on the ventral morphology of this species and compare the available specimens to each other in terms of intraspecific variability. While the overall morphology of *Sinoburius lunaris* has been described in a few works [11, 28], details on the appendages of this species remained unclear until ref. [20] beautifully enlightened the tremendously preserved ventral aspects based on µCT-imaging techniques.

The current most work on *Sinoburius lunaris* [20] described it as being comprised of a cephalon (which we refer to head, see terminology section below) with a semicircular head shield, crescentic in outline, a thorax (= anterior trunk) consisting of seven freely articulating tergites, overlapping each other, and a pygidium of several fused segments. The cephalon bears a pair of small uniramous antennae (= antennulae), which consist of five podomeres (= articles) and an antennal (= antennular) scale they describe as an exite rather than a ramus. Reference [20] wrote of medioventrally located, small, ovoidal, and stalked eyes, which have dorsal exoskeletal bulges as a counterpart, and an ovoidal, elongate and natant (that is, not connected to the shield) hypostome in the head, with a triangular anterior tip. Regarding appendage details, ref. [20] found all three specimens to have 17 biramous post-antennal appendages, with the anterior ones in the cephalon being more gracile than those in the thorax, and all getting smaller towards the posterior end. The cephalon bears four pairs of biramous post-antennal appendages, with the first two having long and stenopodous exopods each consisting of > 12 podomeres, while exopods of the third and fourth pairs of biramous post-antennal appendages share the same morphology as the exopods of the thorax and pygidium. The endopods of the first post-antennal appendages in the cephalon are greatly reduced in size and consist of only five podomeres. On the contrary, the other endopods in the head consist of seven podomeres.

Post-antennal biramous appendages in the thorax are all of the same shape; endopods comprised of seven podomeres with a terminal claw, and exopods made of a slender shaft of two or three podomeres bearing delicate lamellae. The former investigations on *Sinoburius lunaris* [20] mentioned that the size and podomere number of endopods decrease gradually towards the posterior end. Furthermore, Chen et al. [20] also found that the posterior most endopods in the pygidium possess only five observable podomeres, possibly presenting limb buds, that is, not yet fully developed appendages.

Interestingly, as also suggested in previous studies, Chen et al. [20] found a segmental mismatch, that is, a non-correspondence between thoracic tergites and appendages. More precisely, specimen **YKLP 11407** possesses two diplotergites (tergite 4 and tergite 7 with each trunk segment having two pairs of post-antennal biramous appendages instead of only one), whereas the other two specimens, **YRCP 0011** and **Hz-f-10-45** possess only one diplotergite (tergite 7). Hence, the entire number of biramous post-antennal appendages in what they refer to thorax is nine for the first specimen and eight for the other two. For the pygidium, Chen et al. [20] reported three *or* four biramous post-antennal appendages. Eventually, the total number of biramous post-antennal appendages within *Sinoburius lunaris* could not be solved. Some aspects of the morphology, thus, remained puzzling. Besides the problem of segmental mismatch, other things to consider include some appendage details in the anterior body being better preserved in one specimen, while in another specimen posterior appendages reveal more details. Furthermore, the distance from the proximal most preserved article of the antennae to the anterior tip of the hypostome differs between the specimens, making the total length of the antennae questionable.

Taking all this together, we aim to shed new light on a possible intraspecific variability and discuss whether those differences may have resulted from taphonomic processes. We ran volume renderings in Amira and based on them designed 3D models in Blender [27] for each of the three hitherto investigated specimens, that is YKLP 11407, YRCP 0011, Hz-f-10-45 in Chen et al. [20]. Our slice-by-slice tiff stack analyses furthermore include measurements, and we ran a Principal Component Analysis (PCA) based on size-corrected values (via Burnaby-Back-Projection, BBP) of the preserved pygidial appendages to answer the question on having limbs buds (that is, not fully developed appendages).

## Results

As an expansion of and a contribution to the former investigations on *Sinoburius lunaris* [20], our analyses here focus on differences in the preserved morphology within the three specimens. We will not provide an entirely new in-depth description of the morphology of *S. lunaris*, but instead, refer to the reported morphology and focus on specimen-dependent differences. Moreover, we resolve open questions that have not been solved in previous analyses.

### Overall intraspecific variation and preservation of post-antennular appendages

**Fig. 1:**
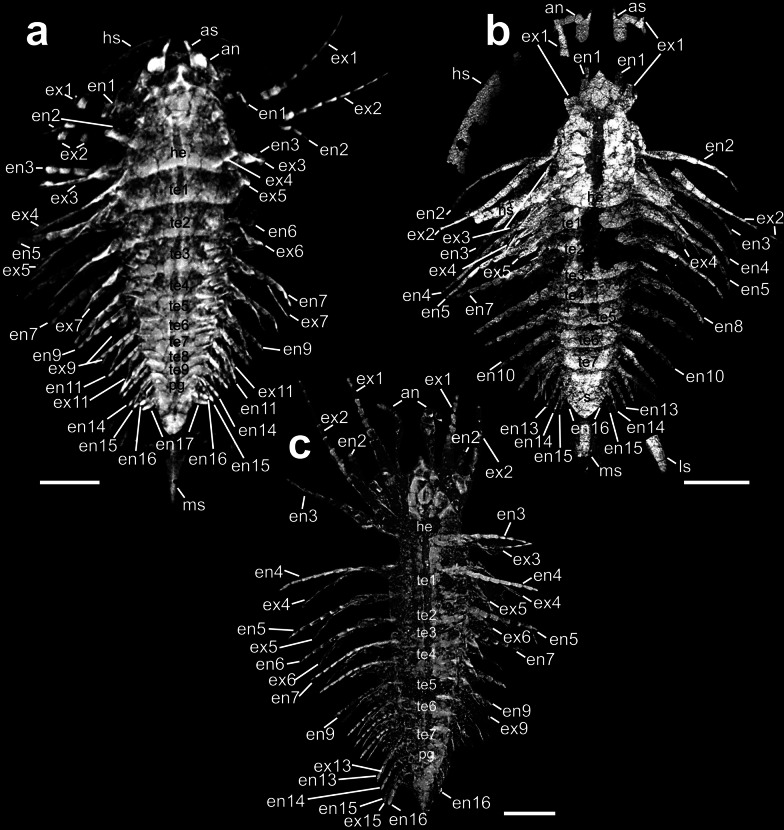
Volume rendering models (MIP, maximum intensity projection) of all three investigated *Sinoburius lunaris* specimens based on X-ray computed tomographic data rendered in Amira, view from dorsal. **a** Specimen YKLP 11407. **b** Specimen YRCP 0011. **c** Specimen Hz-f-10-45. an, antennulae; as, antennular scale; en, endopod; ex, exopod; he, head; hs, head shield piece; ls, lateral spine; ms, median spine; pg, pygidium; te, tergite. Scale bar: 1 mm

**Fig. 2:**
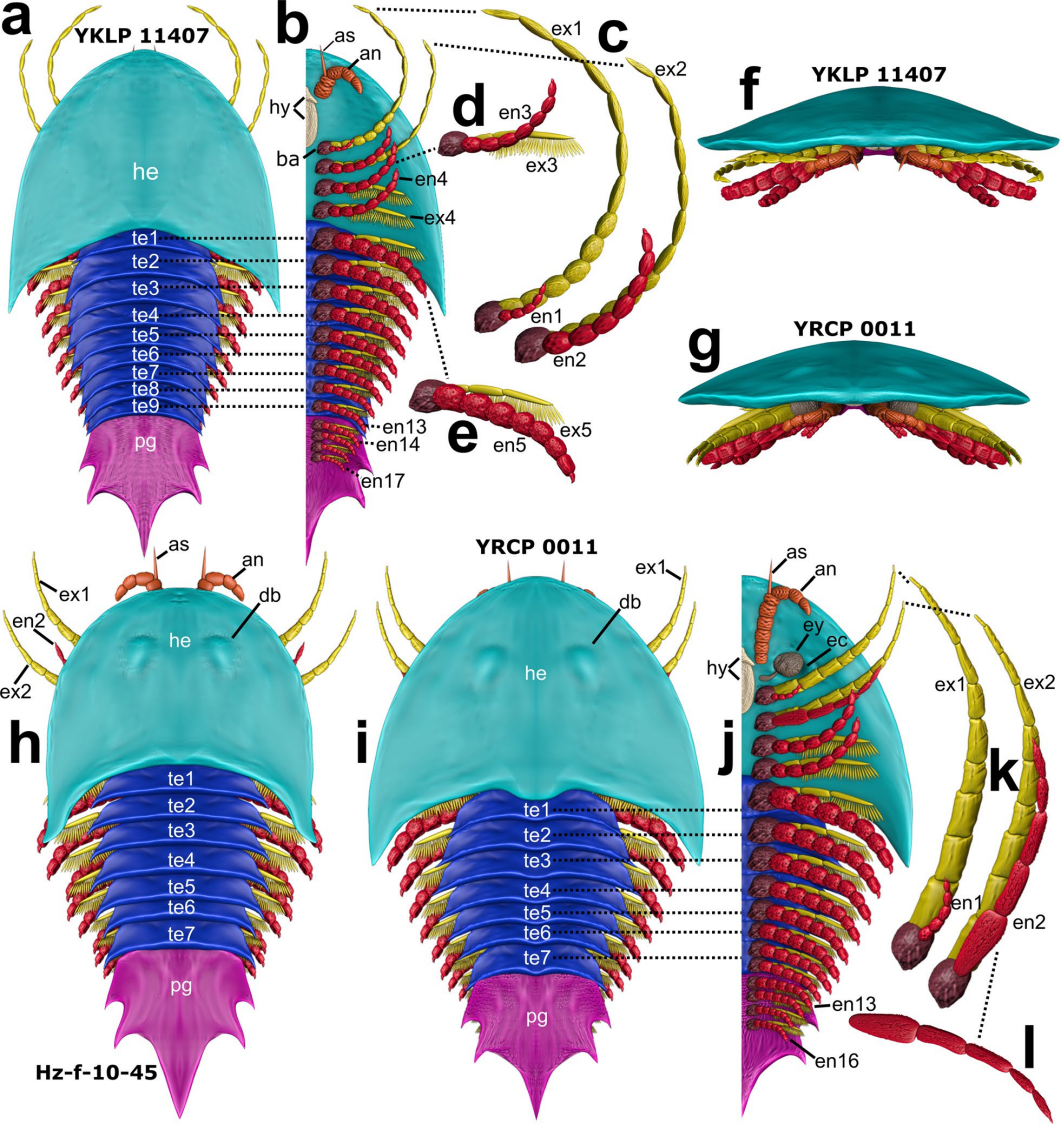
Blender models showing specimen’s shape and appendage details. **a**–**f** YKLP 11407. **g**, **i**–**l** YRCP 0011. **h** Hz-f-10-45. **a**  Dorsal habitus. **b** Ventral habitus, left side. **c** Close-up of appendages 1 and 2. **d** Close-up of appendage 3. **e** Close-up of appendage 5. All posteriorly following appendages are of the same shape. **f** Frontal habitus of specimen YKLP 11407. **g** Frontal habitus of specimen YRCP 0011. **h** Dorsal habitus. **i** Dorsal habitus. **j** Ventral habitus, left side. **k** Close-up of appendages 1 and 2 showing differentiated shape of the exopods. **l** Close-up of endopod 2 pictured as preserved in this specimen (compare Fig. 1b). Appendage numbers count as post-antennular appendages. Note that in **a** no segmental mismatch is pictured, giving nine tergites and nine pairs of appendages, in total 17 post-antennular appendage pairs. In **h**, **i** there are still seven tergites, with te7 as a diplotergite carrying two appendage pairs; 16 post-antennular appendage pairs in total. an, antennulae; as, antennular scale; ba, basipodite; bn, basal most antennular articles; db, dorsal bulges; ec, eye cavity; en, endopod; ex, exopod; ey, eye; he, head; hy, hypostome. Not designed to scale

The volume renderings of all three specimens (Fig. 1) show strikingly delicate appendage structures and enable us to understand the total number of every preserved endo- and exopod. Based on the information given in our Amira volume renderings as well as in the photographs of the specimens presented in the previous study (Fig. 1a, Fig. 4a, Fig. 6a in Chen et al. [20]), we designed 3D models for each specimen and highlighted several appendages. Specimen YKLP 11407 is depicted in Fig. 2a-f, specimen YRCP 0011 is presented in Fig. 2g, i–l. Specimen Hz-f-10-45 (Fig. 2h) is shown dorsally only due to the similarities to specimen YRCP 0011.

#### Body

All three specimens differ slightly in size and show a total length of less than 10 mm. Hz-f-10-45: 8.2 mm, YRCP 0011: 7.5 mm and YKLP 11407: 7.0 mm. For the head shield, we calculated a ratio (width/length). In YKLP 11407 the head shield is more crescentic in outline (ratio 1.69, Fig. 2a), whereas it is more rounded with longer lateral spines in specimen YRCP 0011 (ratio 1.52, Fig. 2i) and narrower in specimen Hz-f-10-45 (ratio 1.31, Fig. 2h). As the latter two specimens do show eyes, we modelled dorsal exoskeletal bulges. While those two specimens bear seven trunk tergites sharing an average length of about 0.36 mm, for specimen YKLP 11407 we count nine tergites (0.4 mm). Of special interest regarding differences in the dorsal morphology is the shape of the medial posterior end of the head shield as well as the shape of the median parts of the tergites. In specimen YKLP 11407 (Fig. 1a, 2a) head shield and tergites are with the concave side oriented anteriorly, whereas in specimen YRCP 0011 they point concave posteriorly (Fig. 1b, 2i). In specimen Hz-f-10-45 they are rather straight (Fig. 1c, 2h). The pygidium measures between 2 and 2.6 mm in all three specimens.

#### Post-antennular head appendages

The post-antennular head appendages of the investigated specimens presumably have a proximal basipodite distally carrying two rami, endopod, and exopod. Specimen YKLP 11407 (Fig. 1a) has mainly the exopods of the right post-antennular appendages 1 and 2 preserved. Specimen YRCP 0011 (Fig. 1b) has no detailed preserved appendage 1 but shows endopods of post-antennular appendages 2–4 in delicately preservation. The former investigations on *Sinoburius lunaris* [20] reported that the endopods of post-antennular head appendages 2–4 have approximately seven articles. Based on the volume rendering, at least the endopod of post-antennular appendage 2 (Figs. 2k, l) is composed of only five articles in this specimen. Those furthermore differ strikingly from each other in shape and length. Endopods of post-antennular appendages 3 and 4 are also elongate and slender, thus differing from the endopods of the trunk and the pygidial appendages. The exact number of articles is not visible, but we illustrated them with seven to be in accordance with all other posteriorly following trunk appendages. Likewise, we modelled endopod 2 of specimen YKLP 11407 with seven articles to be consistent with the assumptions made by the previous study of *S. lunaris*. In Hz-f-10-45, the head shows both first and second elongate stenopodous exopods as well as the third and fourth endopods, but endopods 1 and 2, as well as exopods 3 and 4, are missing. In this specimen, length differences between right and left endopod 3 and endopod 4 are the greatest among all endopods.

#### Trunk appendages

Specimen YKLP 11407 (Fig. 1a) shows eight out of nine preserved endopods as well as exopods in the trunk, making it the most suitable specimen when it comes to reconstructing the original appendage set, at least for the trunk. Specimen YRCP 0011 (Fig. 1b) has all nine trunk endopods preserved, though the right endopod 7 is only fragmentary while the left endopod 6 belonging to trunk segment 2 is absent. Specimen Hz-f-10-45 (Fig. 1c) only has biramous appendages of the left body side well preserved. Furthermore, there are great differences in the total length of the trunk appendages for both sides, as distal articles of the right body side often are not preserved, being biased by taphonomy. As on the left body side endopod 5–endopod 12 are more or less preserved, for the right side only endopod 6–endopod 9 are.

#### Segmental mismatch

The most intriguing finding is that the trunk of specimen YKLP 11407 bears nine tergites instead of seven (Figs. 1a, 2a). This accommodation is visible when following the proximal most articles of the appendages in the volume rendering. Taking this, we can exclude the presence of the two proposed diplotergites (tergite 4 and tergite 7) in this specimen previously attempted by Chen et al. [20] and thus refute the segmental mismatch. This results in a direct correspondence of nine tergites (which form trunk segments dorsally) and nine appendages (Fig. 2b). Otherwise, we agree with the occurrence of the segmental mismatch in the other two specimens YRCP 0011 and Hz-f-10-45 (described in detail in Chen et al. [20], p. 9). Thus, appendage numbers in the trunk for YKLP 11407 refer to appendages 5–13, whereas for the other two specimens, they refer to appendages 5–12. Taking together the four head and the four pygidial appendages (which we will discuss below), this makes up a total of 17 post-antennular appendages in the whole body of specimen YKLP 11407, whereas the other two specimens possess only 16 post-antennular appendages.

#### Pygidial appendages

The pygidium of specimens YRCP 0011 and Hz-f-10-45 were under debate of bearing either three or four biramous appendages ([20], p. 10). We will discuss these preservational circumstances by considering single TIFFs, volume, surface, and isosurface renderings below.

### Antennulae and eyes

Antennulae are preserved in great detail in two specimens, YKLP 11407 and YRCP 0011. They strikingly differ in shape and most of all in position relative to the mouth opening (indicated by the sclerotic plate covering it from anterior, the hypostome). The former investigations on *Sinoburius lunaris* [20] described the shape of the antennulae of specimen YKLP 11407 as preserved bent backwards, indicating a preservation bias, whereas antennulae of specimen YRCP 0011 are preserved rather close to life position. They reported five articles.

**Fig. 3:**
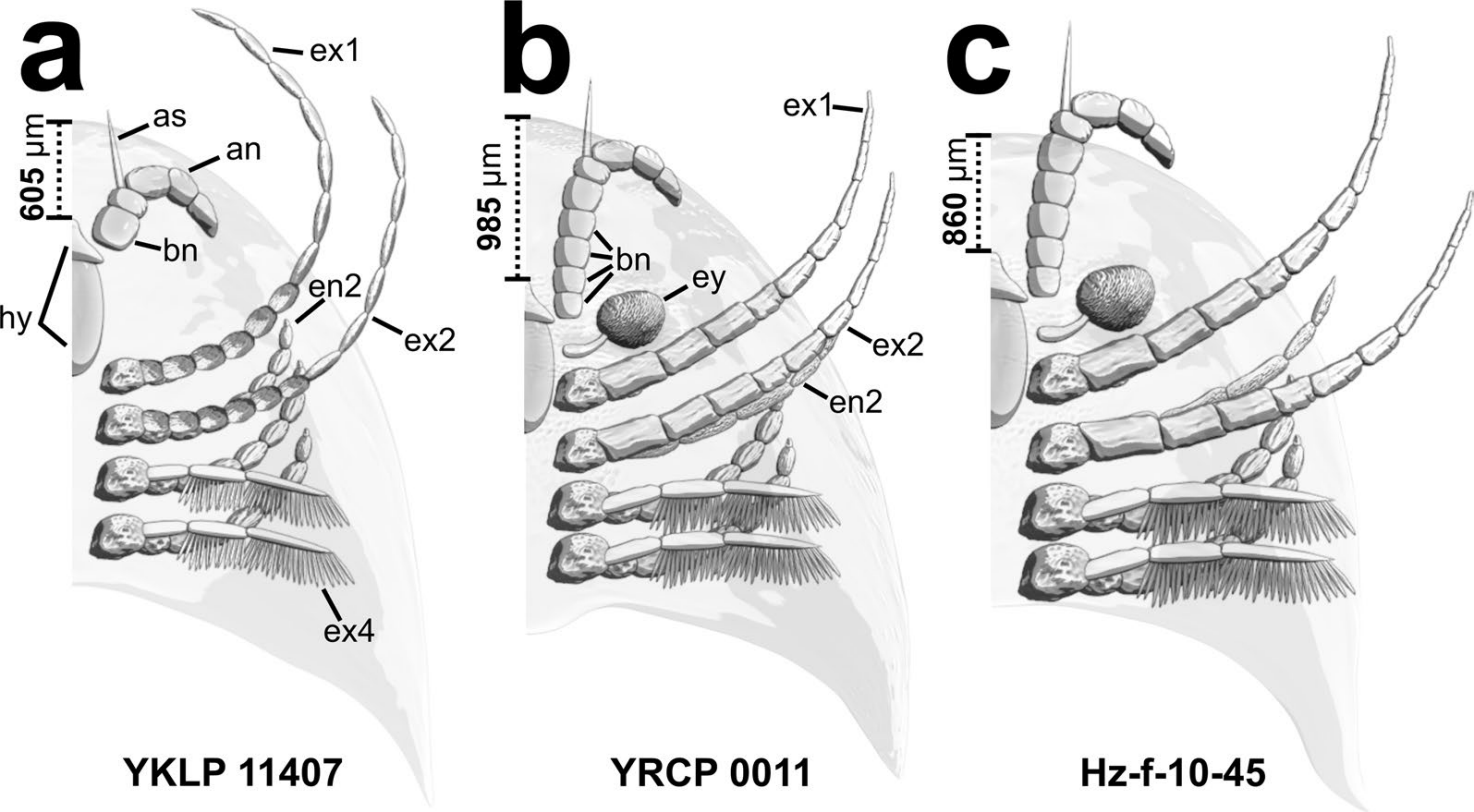
Models showing differences in total distance from the anterior hypostome tip to the anterior margin of the head shield for each specimen. Measurements [µm] were taken directly in Amira. **a** YKLP 11407. **b** YRCP 0011. **c** Hz-f-10-45. View from dorsal with semi-transparent head shield. Head models were built in Blender using Amira volume renderings as well as photographs of the specimens as a template. Length and number of the basal most antennular articles are assumed. Shape and length of the post-antennular head appendages of specimen Hz-f-10-45 resemble those of specimen YRCP 0011 due to the scarcity of appendicular details in the volume renderings of this specimen. an, antennulae; as, antennular scale; bn, basal most antennular articles; en, endopod; ex, exopod; he, head; hy, hypostome. Not designed to scale

**Fig. 4:**
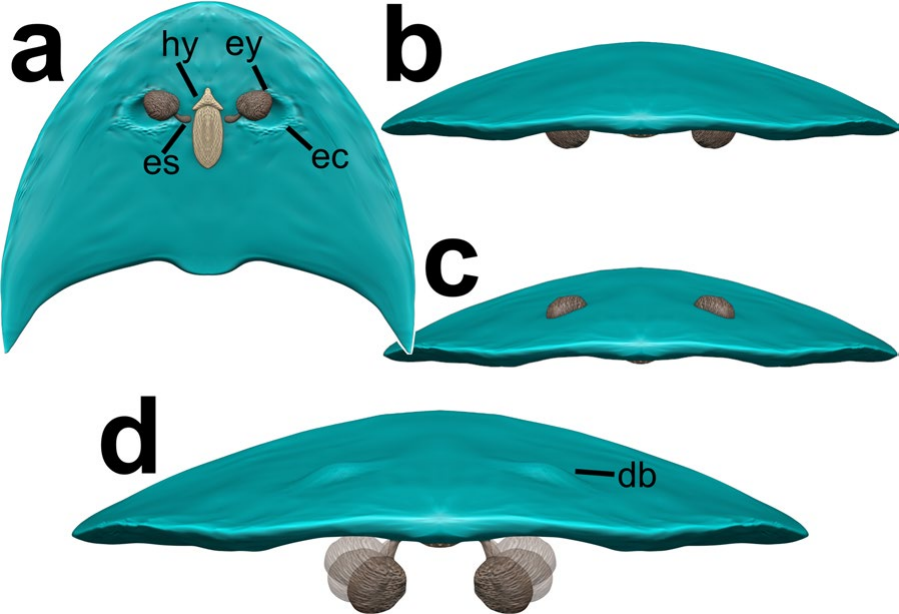
Possible eye locations. **a** Ventral eyes, stalked. **b** Ventral eyes, without stalk. **c** Dorsal eyes, without stalk. **d** Ventral eyes, stalked, mode of eyes swinging-in simulated. **a** Ventral view. **b–d** Frontal view. Models made in Blender based on head shield of specimen YRCP 0011. db, dorsal bulge; ec, eye cavity; es, eye stalk; ey, eye; hy, hypostome. Not designed to scale

The most controversial feature is the distance to the hypostome, the sclerotized plate that covers the mouth opening. Although preserved articles of both specimens slightly resemble each other in length, the distance from the anterior tip of the hypostome to the margin of the head shield measures approximately 985 µm in specimen YRCP 0011 whereas its extension is only 605 µm in YKLP 11407 and 860 µm in specimen Hz-f-10-45. Furthermore, on the microscopic image of specimen YRCP 0011 (see Fig. 4a, in Chen et al. [20], p. 8), elongate structures (impressions) of the same width emerging from the preserved proximal most antennular article 1 to the location next to the hypostome are visible. These were not labelled or mentioned in former studies. To our understanding, this indicates even longer antennulae than previously assumed, thus consisting of even more proximal articles. Taking this into account, those proximal antennular articles must be hardly compressed in specimen YKLP 11407 (Fig. 3).

Though only slightly visible in specimen YRCP 0011 ([20], Fig. 4) and specimen Hz-f-10-45 ([20], Fig. 6), we want to illustrate possible eye locations for this species. The previous study examined “paired exoskeletal bulges accommodating ventral stalked eyes situated mediolaterally” ([20], p. 4). Despite their interpretation, it is not visible, whether they are indeed stalked (and how), and if, whether they indeed are accommodated by dorsal exoskeletal bulges. We, therefore, modelled the head with different interpretations (Fig. 4). Given ventral stalked eyes, a groove seems to be of advantage to swing them in for protection. This might indeed result in the accommodation of dorsal bulges (Figs. 4a, d). Otherwise, they could have been also unstalked (Fig. 4b, c). A mode not shown but also possible is nowadays present in notostracans like *Triops* sp. or *Lepidurus* sp., where the dorsally located nauplius eye can look down to the bottom through their carapace due to transparent window-like structures in their cuticle [29].

### In-depth analyses of the appendages in the pygidium

**Fig. 5:**
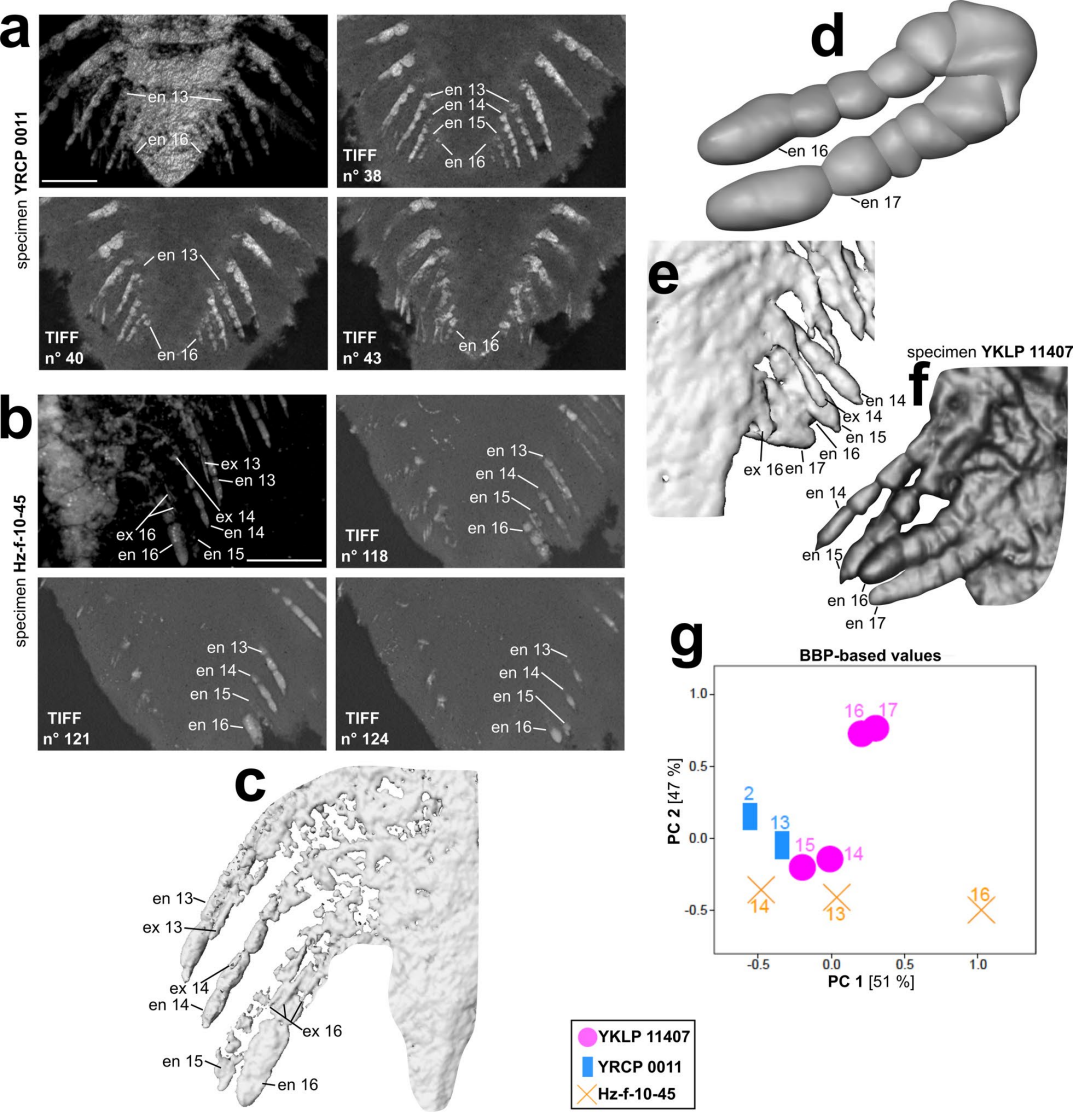
In-depth analyses of the pygidial appendages of all three investigated specimens of *Sinoburius lunaris*. **a** Volume rendering and TIFF slices no 38, 40 and 43 of specimen YRCP 0011, dorsal view. **b** Volume rendering and TIFF slices no 118, 121 and 124 of the left pygidial appendages of specimen Hz-f-10-45, ventral view. **c** Isosurface model showing remnants of the left endopods and exopods of appendages 13–16 of specimen Hz-f-10-45, dorsal view. **d** Surface model of endopod 16 and 17 of specimen YKLP 11407 with prospective article boundaries (basipodite not possible to reconstruct due to preservation). **e** Isosurface model showing remnants of the right endopods and exopods of appendages 14–17 of specimen YKLP 11407, dorsal view. **f** Volume rendering (VRT) of the right pygidial appendages of specimen YKLP 11407, ventral view. **g** PCA plot of the pygidial appendages of all specimens. Each symbol represents one appendage (mean length of right and left appendages). Values were size-corrected via Burnaby-Back Projection (BBP). Eigenvalues: PC1: 0.235114. PC2: 0.217857. Renderings done with Amira. en, endopod; ex, exopod

The previous study ([20], p. 10) assumed three or four appendages under the pygidium as Drishti volume renderings showed no clear results on that precise question. Given the segmental mismatch in specimens YRCP 0011 and Hz-f-10-45, the first pair of post-antennular appendages under the pygidium are represented by appendage number 13, whereas in YKLP 11407 it is number 14. To enlighten those vague appendicular aspects of the pygidium, we combined several visualization methods (Fig. 5).

The volume rendering of specimen YRCP 0011 indicates at first sight only three visible pygidial appendages (Fig. 5a). We thus made use of the single TIFF images of the µCT scans. For specimen YRCP 0011, 2D TIFF slice no 38 shows endopods 13–15 with four to six articles countable. Near endopod 15, the first hint of a possible 16th endopod (making it the 4th pygidial endopod) is given. In 2D TIFF slice no 40, left endopod 16 is presented, showing two articles. Ultimately, 2D TIFF slice no 43 shows three visible articles of right endopod 16 (or even four when looking at the distal most part of this endopod).

For specimen Hz-f-10-45 (Fig. 5b), only the left pygidial appendages are preserved. Here, likewise only three pygidial appendages are visible. This specimen shows endopod 16 in delicate detail, which differs to a great extent from the former endopods according to its width. In contrast to specimen YRCP 0011, very slender structures are preserved next to endopods 13–15 in specimen Hz-f-10-45. We interpret them as being the corresponding exopods (Fig. 5c).

For specimen YKLP 11407, Chen et al. [20] already described four pygidial appendages, (post-antennular appendage numbers 14–17). As the last two pygidial endopods are best preserved in this specimen and of a different shape than those of the others, we created a surface model based on their volume and isosurface renderings (Fig. 5d). The basipodite is not clearly visible and the proximal most articles are small and compressed. The distal most articles otherwise are elongate. We can count up to five articles, which are also visible in the former two endopods (endopod 14 and 15, Fig. 5f) and consistent with the suggestions in Chen et al. [20]. They thought endopod 16 and 17 may represent rather limb buds rather than fully grown biramous appendages but were not able to support this assumption. The former study [20] did not provide any precise definition of limb buds. We use this term to differentiate between the shape of those rather dumpy and wider endopods and all other endopods, having equally shaped articles. Thus, we define limb buds as not fully-grown appendages, being still in development, which we infer from the shape of their articles in comparison to the shape of the articles of all other endopods we believe to be fully developed.

The distal most article in endopod 16 of specimen Hz-f-10-45 (Fig. 5c) slightly resembles the distal most articles shown in endopod 16 and 17 of specimen YKLP 11407 (Fig. 5d), that is being thicker, elongate, and more roundish and finger-shaped than the others. Another aspect might be the terminal claw, which is developed for endopod 14 and 15, but not for endopod 16 and 17 (Fig. 5f), given that the development of a terminal claw represents a fully-grown leg. Comparing those two specimens with their finger-shaped distal most articles, we can pay attention to the development of the exopods.

In specimen Hz-f-10-45, the remains of a presumably developed exopod are preserved in terms of filamentous structures (exopod 16, Fig. 5c), whereas specimen YKLP 11407 only shows remnants of a probable beginning development of exopod structures (Fig. 5e). Taking all this information together we can conclude the following:

1. The last two pygidial appendages of specimen YKLP 11407 (= endopod 16 and 17) appear to be limb buds, thus not yet fully developed limbs.
2. The last two pygidial appendages of specimen Hz-f-10-45 do represent further developed biramous appendages in terms of having a developed exopod on the last pygidial appendage and a nearly completely developed penultimate pygidial appendage.

We are not able to say whether the last pygidial appendage of specimen Hz-f-10-45 is a fully developed biramous appendage by looking at the finger-shaped distal most article, which resembles those in specimen YKLP 11407. Both could hint on article differentiation. Nevertheless, we can conclude that those last two pygidial appendages in specimen YKLP 11407 are in a less developed state in comparison to those in specimen Hz-f-10-45.

This is also shown in the PCA we ran for all pygidial appendages that were appropriate for measuring (Fig. 5g). PC1 explains 51% of the total variance, while PC 2 explains 47% of the total variance. All PCA-related data as well as underlying article measurements are summarized in Additional file 1: Table S1. This PCA plot represents, on the one hand, the clustering of all preserved pygidial appendages, on the other hand, it portrays endopod 2 of specimen YRCP 0011, which is the most modified endopod of all specimens (see also Fig. 2l). The measured biramous post-antennular appendages in the pygidium were endopod 14–17 in specimen YKLP 11407 (purple dots), endopods 13, 14 and16 in specimen Hz-f-10-45 (orange cross) and endopod 13 in specimen YRCP 0011 (blue square).

While the endopods 13–15 of all specimens group together, the very last appendages of specimen YKLP 11407 form a distinct cluster—distant to endopod 16 of specimen Hz-f-10-45.

While endopods 16 and 17 of specimen YKLP 11407 appear at positive PC 2 values, endopod 16 of specimen Hz-f-10-45 is rather close to the other, fully differentiated endopods (though slightly separated) at negative PC2 values. Though the sample size of this analysis is quite low, the clustering confirms our interpretation above that the endopod 16 of specimen Hz-f-10-45 is in a further developed state than endopods 16 and 17 of specimen YKLP 11407.

## Discussion

In terms of an improved understanding of the ventral morphology, we focused on the overall shape of the specimens, the number of tergites, the composition of the antennulae, and the number of pygidial appendages in general and with special regard to possible limb buds. We compare our results with the recent morphological understandings of *Sinoburius lunaris* made by Chen et al. [20]. Additionally, we want to draw attention to previous morphological understandings and misconceptions.

### The understanding of the morphology of Sinoburius lunaris and its change through time

Up to now, seven specimens of *Sinoburius lunaris* have been mentioned in literature. In addition to the holotype (NIGPAS Cat. No. 115287) and the paratype (NIGPAS Cat. No. 115288) ([28], Fig. 4; [11]), further figured specimens are ELRC 19550 ([30], Fig. 215; [31]), as well as ELRC 19551 ([31]; [32], Figs. 88, 89). The herein considered analyses of [20] contributed three additional specimens (YKLP 11407, YRCP 0011, and Hz-f-10-45), whilst the latter one had already been presented in Ref. ([33], Pl. II, Fig. 4).

Thus, this species is still rare, and detailed morphological analyses before Chen et al. [20] used 'traditional methods' such as light microscopy and needle preparation, resulting in different morphological observations.

The original description of *Sinoburius lunaris* [28] considered the holo- and the paratype. Not much known was about the appendage morphology. The head was assumed to have a pair of antennulae (originally termed 'antennae') followed by three or four pairs of additional post-antennular head appendages [11]. The antennulae were drawn as being composed of numerous articles ([11], Figs. 78c, 79). This was assumed due to the presence of shallow furrows on the head shield in the posterior part of the paratype. Later, the same specimen (Hz-f-10-45) was re-figured [33] which was investigated in both, Chen et al. [20] and our study. 'Two pairs of antennae' instead of a single pair were also reported [33]. They mentioned a supposedly ´interior` pair (i.e., the inner ones) being smaller ([33], p. 130). If we compare this statement to our understanding of the morphology of specimen Hz-f-10-45 (see Figs. 1c, 2h, 3c herein; consider also Fig. 6 in [20]), we can assume that [33] misinterpreted the first pair of post-antennular exopods as a kind of 'exterior antennae', while their reference to the inner' ones represented the antennulae. The interpretation of [33] is especially interesting, as [11] already identified the antennulae and recognized the supposed 'exterior' ones as parts of a biramous appendage (in this case, however, they thought of endopods; see *Len1* in ([11], Fig. 78b).

The antennulae in *Sinoburius lunaris* according to Chen et al. [20] consist of five observable articles. Nevertheless, Figs. 6a, b in [20] indicate that the antennulae indeed might have been even longer, considering the furrows on the slab. In the earlier studies, the illustration of the head region of this specimen does not seem to represent the original length of the antennulae ([11], Fig. 79). The so-called 'antennal scale' (see [20], p. 2), however, might have been a filament-like structure and much longer than preserved, possibly also protruding under the head shield like the antenniform first exopods. But there is no confirmation of their original length. Regarding the trunk, each of the seven tergites formed by the respective body segments was thought to possess one pair of biramous appendages due to visible furrows [11]. The former investigation on *S. lunaris* [20] demonstrated that two tergites correspond to more than one pair of appendages in specimen YKLP 11407 (tergite 4, tergite 7) and one in Hz-f-10-45 (tergite 7), making up eight respectively nine pairs of trunk appendages. We refute the presence of diplotergites at least for specimen YKLP 11407, resulting in nine tergites, each corresponding to one pair of biramous post-antennular appendages. Another morphological structure that has caused controversy is the pygidium. This was once assumed to be composed of at least ten segments with the anterior six bearing biramous appendages ([11], p. 2). This conclusion was drawn based on the posterior part of the holotype NIGPAS Cat. No. 115287 (see Figs. 77a, 78a in ref. [11]). The previous investigation on *Sinoburius lunaris* [20] contrasted with the assumption of three *or* four pairs of pygidial appendages. Yet, due to the limitations of preservation neither volume nor surface renderings could provide clear results. Only via comparisons of 2D slices, we are able to show that there are indeed four pairs of pygidial appendages in all three analyzed specimens.

### Specimen-dependent morphological differences

The way representatives of Euarthropoda are segmented and the overall meaning of segmentation has long been discussed [34–37]. A direct match between body segments (or rather dorsal and ventral sclerites) and appendages is the most common mode with one pair of appendages belonging to one body segment. Contrarily, a segmental mismatch describes a discordance between those sclerites of tergites and sternites. In some extant representatives of Euarthropoda, there is a high variability with, for instance, symphylans and some centipedes having more dorsal sclerites than pairs of trunk appendages. Vice versa, pauropods, and millipedes have fewer tergites than pairs of trunk appendages [38, 39]. Also, segments possessing more than one pair of appendages occur in notostracans [40].

For *Sinoburius lunaris*, segmental mismatch was also demonstrated [20]. According to  its  former analysis [20], two specimens (YRCP 0011 and Hz-f-10-45) had 16 pairs of biramous post-antennular appendages and seven tergites in the trunk (counting for seven distinguishable trunk segments) with only the seventh trunk segment bearing two appendage pairs. This we could also confirm. For the other specimen (YKLP 11407), a total of 17 post-antennular biramous appendages was found, also seven trunk segments but with trunk segment four and seven each carrying two pairs of biramous appendages [20].

We could enlighten this discordance, as we found specimen YKLP 11407 having rather nine than seven trunk segments–giving that one trunk segment is forming one tergite dorsally. However, there is still inconsistency between the three specimens of *S. lunaris*, that said given the variability of tergites, and possible diplotergites or syntergites.

This could be due to several reasons. It might be just a case of intraspecific variability, making this species highly variable concerning major morphological features. In extant notostracans [40], the number of biramous trunk appendages can vary greatly, and for epimorphic centipedes and adesmatan geophilomorphs, high variability in segment numbers within one species has also been described [41, 42]. However, the variability in segment numbers in some centipedes should not be confused with a dorso-ventral mismatch of segmental structures.

Sexual dimorphism could be another case to take into consideration and also explain not only the different number of trunk segments (dorsally forming tergites) but also the different number of total appendages within all three specimens. In some extant polydesmidan millipedes and adesmatan centipedes, females possess more segments than males [39], whereas in some notostracans this is the other way around [40]. Additionally, for the latter group, even within-sex intraspecific variability is described, with males having 38–44 leg-bearing trunk segments [40], see also survey in ref. [36].

A third scenario may be shown by all *Sinoburius lunaris* specimens representing different ontogenetic stages. Thus, an anamorphic development could be addressed. This implies that segmental units are added during post-embryonic ontogeny, as it is found in many crustaceans, but also in proturan insects [43] and many myriapods (e.g., compare survey in ref. [44]). Furthermore, also for trilobites [45–47] and megacheiran Cambrian arthropods [18], this developmental pattern is described, following an anterior–posterior developmental gradient.

The total size of the investigated specimens, however, may refute this idea, as the smallest specimen is YKLP 11407, being also the one with the higher number of both tergites and total post-antennular appendages. However, the size of post-embryonic ontogenetic stages of arthropods depends also on food and temperature [48]. Thus, YKLP 11407 can be the most advanced developmental stage despite being the smallest individual specimen, like given the ventral parts of segments develop at a faster pace compared with the dorsal parts [46]. Overall, the range of total body size within the three specimens is not that high. The question for the reason of the morphological inconsistency may finally only be entangled with a higher number of investigated specimens of different total body sizes.

A last scenario might be given if the three investigated specimens would belong to more than just the one described *Sinoburius lunaris* species. At least the differences in the shapes of the head shields, the tergites, and the head appendages between YKLP 11407 and YRCP 0011 suggests this. Again, a wider taxon sampling of different body sizes in the future could shed light on this aspect.

### Amira vs. Drishti in the light of virtual palaeontology

Both, Amira [49, 50] and Drishti [26] provide a useful software to process µCT data on extinct and extant arthropods and to visualize certain aspects of their morphology. Together with other programs like MeshLab or Blender, one can visualize its µCT-generated models in a variety of ways [51].

While Amira is a single program containing a delightful set of volume and surface rendering modes, Drishti comes with three distinct programs (Drishti Import, Drishti Paint and Drishti, the renderer itself). Both, Amira and Drishti possess a diverse range of user-friendly options to work in 3D on the volume models as well as in 2D on the single TIFF slices. The biggest advantage of Amira might be the opportunity to directly process a surface reconstruction based on the segmentation of individual structures. Those surface models later can be exported to use in other 3D modelling programs like Blender or Autodesk Maya in terms of a kinematic approach [24]. Nevertheless, this surface reconstruction method is also feasible in Drishti Paint. Besides surface reconstruction, both programs also offer a great anmount of volume rendering tools.

We reinvestigated the three *Sinoburius lunaris* specimens with Amira in order to make advance of its different volume rendering settings *VRT* and *MIP*. *VRT* is a texture-based volume rendering with different shading options like *Diffuse* or *Specular*. *Diffuse VRT* sets a diffuse light source, whereas the *Specular VRT* option offers a simulation of a specular visualization of the specimen. The latter one may most likely resemble the pre-sets of Drishti volume rendering making the objects look more vivid. The *MIP* (maximum intensity projection) volume rendering mode otherwise displays the brightest data value along each ray of sight instead of showing the result of the emission absorption model. This makes it possible to look through the fossil and detect for example underlying structures. Hence, we used this volume rendering mode for the visualization of the entire specimens (Fig. 1), whereas we used the *VRT* mode the get a more vivid and plastic look of the appendages (Fig. 5f). We think, in the near future fossils could–in the light of virtual paleontology–benefit from a variety of 3D visualization and modelling programs to explore their morpho-functionality in many different ways.

### Applicability of morphometrics to analyses of Chengjiang arthropods

This is the first study in which a PCA based on Burnaby-Back Projection size-corrected data was run when analyzing Chengjiang arthropods.

A PCA is the most abundant and reliable multivariate method to ordinate data but has essential preconditions, one being normally distributed data. Testing for normal distribution makes no sense regarding a sample size of only three specimens. However, according to the Central Limit Theorem [52]—which states that the sampling distribution of the sample average approximates a normal distribution as the sample size gets larger—a calculation of a PCA is possible, though.

Speaking of large sample sizes, the question of the minimum number of variables in a PCA calculation is another topic [53, 54], and so is the bias of the resulting plots considering outliers. Our analysis could, of course, be enhanced by a larger set of specimens of *Sinoburius lunaris*, resulting in more clusters to compare. A statistically more comprehensive analysis could be done considering all post-antennular biramous appendages. This was not possible due to the fact that a BBP only works with the same number of articles, and we could only measure five articles in the pygidium. Thus, we could not include size-corrected BBP data for endopods with seven articles in this PCA, which all other anterior most appendages bear.

Size-correction is a pivotal precondition when working with metric data. Otherwise, analyses would just show size-dependent patterns, hence skewing the real information. The most common size-reduction method is creating ratios, i.e., dividing the measured body parts through the body length of the respective specimen or species. Despite its common use, several authors have criticized such data still being size-dependent [55–57].

Another widely used method, as said, is size-correction via the so-called Burnaby-Back Projection. The theory of this method was introduced in 1966 [58] and further developed by [59]. The BBP size-correction model, in the end, provides the information of the location of a data point in space and its relative position to all other points while neglecting the assumed growth vectors created for all of those data points (for a further description, see [60, 61]). For this reason, we favored this size-correction method.

Furthermore, it is crucial to work with missing values. If any article in the middle of an endopod is missing, i.e., it is not preserved in a good way to measure, then there are several ways to handle this problem, like iterative computation. This might work also for missing terminal articles—but only, if they were not preserved. In our data, we could count seven articles for the post-antennular biramous head and trunk appendages (despite endopod 2 of specimen YRCP 0011), while pygidial appendages only showed five. For this arrangement, a complete PCA of the entire data set is not possible, and neither a BBP is, as mentioned above.

## Conclusions

We re-visited the morphology of *Sinoburius lunaris* and attained a deeper and more comprehensive understanding of the ventral morphology also paying attention to specimen-dependent differences. In further analyses, multivariate statistics, as well as morphometrics, can be applied considering more specimens. Body, endopod, and article measurements, such as length and width might be taken into consideration to look for patterns and changes among all specimens regarding all endopods from anterior to posterior in terms of a morphometric approach. Lots of new fossils are excavated in the Chengjiang region continuously, hence there might soon appear additional specimens with delicate structures preserved well enough for scanning.

## Methods

We worked on the original datasets derived from µCT-scans of the following fossils of *Sinoburius lunaris*: YKLP 11407, YRCP 0011, and Hz-f-10-45, all previously analyzed in Chen et al. [20]. Information about fossil collection and housing is specified therein.

### Scanning

According to [20], the scans of specimen YKLP 11407 and specimen Hz-f-10-45 were performed with a Zeiss X-radia 520 Versa (voltage: 81 kV, current: 50 μA); specimen YRCP 0011 was scanned with a GE Phoenix Nanotom (voltage: 110 kV, current: 100 μA). We used the original TIFF stacks of all three specimens for our 3D anatomical investigations.

### Amira—volume renderings

We processed the TIFF stacks with the commercial software package Amira 6.3 (FEI Visualization Sciences Group, Zuse Institut, Berlin; see ref. [49, 50]). Volume renderings were figured in *MIP* and *VRT* mode.

### Blender—3D modelling

3D models were created in Blender 2.9 [27] using simple meshes like spheres and cubes which were shaped with the sculpting tool.

### Morphometric analyses

We measured head, trunk, and pygidium at the isosurface models in Amira using the digital ruler tool. Furthermore, we took measurements of the endopods of the pygidial appendages and their articles of all specimens for both left and right appendages. To reduce the effect of taphonomic biases, we calculated with the average. We transformed the absolute values to relative values in order to reduce the size effect via the so-called Burnaby Back Projection (BBP; [58, 59]) and ran a PCA. Due to the low sample size, we did not test for normal distribution. Though this is in general a prerequisite when calculating a PCA, we refer to the Central Limit Theorem [52, 62]. Multivariate analysis of the pygidial appendages, as well as the resulting graph and the Additional file 1: Table S1 was done in PAST 3.26 (https://folk.uio.no/ohammer/past/; PALaeontological STatistics). Size-correction via BBP was calculated in R (https://www.r-project.org/; packages: readxl, MAAS), based on the R-script written by [63], see also ref. [64].

### Terminology

We use the terms summarized in [11, 65], following [66] as well as [67, 68]. The terms *cephalon*, *antennae*, *antennal scale*, *thorax*, and *podomeres* were listed in the previous study on *Sinoburius lunaris* [20]. For a more comprehensive understanding and to avoid unfortunate coincidence with the malacostracan terminology, we refer to them as *head*, *antennulae*, *antennular scale*, *trunk*, and *articles*. Although trunk in general is meant to imply everything following the head posteriorly, we use this term to refer to the body part between the head and the pygidium, thus using it equivalently to the term thorax in Chen et al. [20]. Furthermore, although [11] use the term *antennae* for all fossil arthropods discussed there, we speak of *antennulae* as deriving from the second head segment (thus being deutocerebrally innervated).

***Antennulae:*** anterior most appendages, usually uniramous, deutocerebrally innervated.

***Articles: ***elements an appendage is comprised of.

***Basipodite***: proximal most part of an appendage, giving rise to endopod and exopod.

***Head: ***segments dorsally forming a shield, possibly a synsclerite formed by the segments of the anterior most tagma.

***Hypostome***: a sclerotized plate covering the mouth opening.

***Endopod***: inner branch of a biramous leg, arising from the basipodite.

***Exopod:*** outer branch of a biramous leg, arising from the basipodite.

***Pygidium:*** posterior tagma of the trunk, recognizable by a distinct large syntergite continuous with the telson.

***Trunk:*** body region posterior to the head.

***Tergite:*** sclerotized plate on the dorsal side of the animal.

## Supplementary Information

**Additional file 1: Table S1.** SM1 Underlying data of Principal Component Analyses regarding all endopods with five articles. Mean length of left and right endopod articles [μm], BBP size-corrected values of mean length of left and right endopod articles, PCA scores and PCA loadings.

## Data Availability

The original material of *Chen X, Ortega-Hernández J, Wolfe JM, Zhai D, Hou X-G, Chen A, Mai H, Liu Y. The appendicular morphology of* Sinoburius lunaris *and the evolution of the artiopodan clade Xandarellida (Euarthropoda, early Cambrian) from South China. BMC Evolutionary Biology.2019;19:165* was used. The material is deposited in the Yunnan Key Laboratory for Palaeobiology, Yunnan University (YKLP 11407), Yuxi Normal University (YRCP 0011), and Yunnan Institute of Geological Survey (Hz-f-10-45). **Declarations**

## Abbreviations

an: Antennulae
as: Antennular scale
ba: Basipodite
bn: Basal most antennular articles
db: Dorsal bulges
ec: Eye cavity
en: Endopod
es: Eye stalk
ex: Exopod
ey: Eye
he: Head
hs: Head shield piece
hy: Hypostome
ls: Lateral spine
ms: Median spine
pg: Pygidium
te: Tergite
