# Supplementary material for: Intraspecific variation in the Cambrian: new observations on the morphology of the Chengjiang euarthropod Sinoburius lunaris
Source: BMC Ecol Evol. 2021 Jun 21;21:127. doi: 10.1186/s12862-021-01854-1 (PMC8215796; doi:10.1186/s12862-021-01854-1)
Supplement: Supplementary file 1 — Additional file 1: Table S1. SM1 Underlying data of Principal Component Analyses regarding all endopods with five articles. Mean length of left and right endopod articles [μm], BBP size-corrected values of mean length of left and right endopod articles, PCA scores and PCA loadings. [file 12862_2021_1854_MOESM1_ESM.docx]

**TAB. SM1** Underlying data of Principal Component Analyses regarding all endopods with five articles. Mean length of left and right endopod articles [um], BBP size-corrected values of mean length of left and right endopod articles, PCA scores and PCA loadings.
